# Trends, determinants and differences in antibiotic use in 68 residential aged care homes in Australia, 2014–2017: a longitudinal analysis of electronic health record data

**DOI:** 10.1186/s12913-020-05723-3

**Published:** 2020-09-18

**Authors:** M. Z. Raban, K. E. Lind, R. O. Day, L. Gray, A. Georgiou, J. I. Westbrook

**Affiliations:** 1 Centre for Health Systems and Safety Research, Australian Institute of Health Innovation, Macquarie University, Level 6, 75 Talavera Rd, Macquarie Park, Sydney, NSW 2109 Australia; 2 Department of Health Promotion Sciences, Mel & Enid Zuckerman College of Public Health, University of Arizona, 3950 S. Country Club Rd., Suite 330, Tucson, AZ 85714 USA; 3 Department of Clinical Pharmacology & Toxicology, St Vincent’s Hospital, Therapeutics Centre, Level 2 Xavier Building, St Vincent’s Hospital, Darlinghurst, Sydney, NSW 2010 Australia; 4 St Vincent’s Clinical School, UNSW Medicine, University of New South Wales, Level 5 deLacy Building, St Vincent’s Hospital, Victoria St, Darlinghurst, Sydney, 2010 NSW Australia; 5 Centre for Health Services Research, The University of Queensland, Level 2, Building 33, Princess Alexandra Hospital campus, Woolloongabba, Brisbane, 4102 QLD Australia

**Keywords:** Anti-bacterial agents, Long-term care, Residential facilities, Nursing homes, Homes for the aged, Electronic health records, Pharmacoepidemiology

## Abstract

**Background:**

Internationally, point prevalence surveys are the main source of antibiotic use data in residential aged care (RAC). Our objective was to describe temporal trends in antibiotic use and antibiotics flagged for restricted use, resident characteristics associated with use, and variation in use by RAC home, using electronic health record data.

**Methods:**

We conducted a retrospective cohort study of 9793 unique residents aged ≥65 years in 68 RAC homes between September 2014 and September 2017, using electronic health records. We modelled the primary outcome of days of antibiotic therapy /1000 resident days (DOT/1000 days), and secondary outcomes of number of courses/1000 days and the annual prevalence of antibiotic use. Antibiotic use was examined for all antibiotics and antibiotics on the World Health Organization’s (WHO) Watch List (i.e. antibiotics flagged for restricted use).

**Results:**

In 2017, there were 85 DOT/1000 days (99% CI: 79, 92), 8.0 courses/1000 days (99% CI: 7.6, 8.5), and 63.4% (99% CI: 61.9, 65.0) of residents received at least one course of antibiotics. There were 7.7 DOT/1000 days (99% CI: 6.69, 8.77) of antibiotics on the WHO Watch List administered in 2017. Antibiotic use increased annually by 4.09 DOT/1000 days (99% CI: 1.18, 6.99) before adjusting for resident factors, and 3.12 DOT/1000 days (99% CI: − 0.05, 6.29) after adjustment. Annual prevalence of antibiotic use decreased from 68.4% (99% CI: 66.9, 69.9) in 2015 to 63.4% (99% CI: 61.9, 65.0) in 2017, suggesting fewer residents were on antibiotics, but using them for longer. Resident factors associated with higher use were increasing age; chronic respiratory disease; a history of urinary tract infections, and skin and soft tissue infections; but dementia was associated with lower use. RAC home level antibiotic use ranged between 44.0 to 169.2 DOT/1000 days in 2016. Adjusting for resident factors marginally reduced this range (42.6 to 155.5 DOT/1000 days).

**Conclusions:**

Antibiotic course length and RAC homes with high use should be a focus of antimicrobial stewardship interventions. Practices in RAC homes with low use could inform interventions and warrant further investigation. This study provides a model for using electronic health records as a data source for antibiotic use surveillance in RAC.

## Background

The World Health Organisation (WHO) has declared antibiotic resistance one of the greatest threats to global health [1]. Widespread and inappropriate human use of antibiotics is a leading contributor to antibiotic resistance. To address inappropriate antibiotic use it is important to understand antibiotic use not only at the population level, but also in specific sub-populations. Antibiotic use in residential aged care RAC), also known as nursing homes and long-term care facilities, is higher than in the general population and studies report that approximately 50% of courses in this setting are inappropriate [2–4].

National population level antibiotic consumption in Australia is above the OECD average [5]. However, after recording steady increases in national antibiotic consumption between 2013 and 2015, the Antimicrobial Use and Resistance in Australia (AURA) surveillance program reported a continuing downturn in antibiotic consumption beginning in 2015 [5]. However, it is unknown whether antibiotic use in RAC in Australia has also decreased as it has at the national level.

Point prevalence surveys have been implemented internationally to measure antibiotic use in RAC and are currently the main source of data in this setting. In Australia, there have been three large point prevalence surveys conducted annually as part of AURA [5, 6]. The reported percentage of residents using systemic antimicrobial agents was 7.5%, 6.7 and 6.7% on a single day in 2016, 2017, and 2018, respectively [5, 6]. Point prevalence surveys provide a snapshot of antibiotic use, but they cannot assess seasonal trends, and have limited capacity to characterise longitudinal trends in antibiotic use. Additionally, point prevalence surveys can be resource-intensive requiring chart review by staff of already under-resourced RAC homes [7]. The implementation of electronic health records in RAC provides a new opportunity to examine antibiotic use in RAC in more detail, while reducing reporting burden on RAC staff. A number of studies have demonstrated the utility of these data but electronic health records are still underutilised for monitoring purposes [8–10].

The aim of this study was to describe and evaluate the trends in antibiotic use in Australian RAC using electronic health record data. Specifically, the objectives were to describe temporal trends in overall antibiotic use and antibiotics flagged for restricted use, and to examine RAC home variation and resident characteristics associated with antibiotic use.

## Methods

### Data

Data were extracted from the electronic health records of residents in RAC homes run by of a large not-for-profit aged care provider in New South Wales and the Australian Capital Territory. The dataset included resident demographics, stay information (dates admitted and discharged), free text fields containing resident health conditions, Aged Care Funding Instrument (ACFI; an assessment of health and functional status) records, and details of all medications administered (medication name, date and time of administration). Historical data were extracted in September 2017.

### Sample

We limited our sample to permanent residents (i.e. not users of respite care) of 68 RAC homes, who were aged≥65 years with a minimum length of stay of two weeks. Residents enter and exit the sample at different times depending on when they are admitted or discharged from the RAC home, which made this a retrospective dynamic cohort study. The electronic medication administration system was rolled out across the RAC homes throughout 2014. Since all homes had implemented the system by September 2014, we chose this as the start point for measuring antibiotic use.

### Identification of antibiotic medications

The medication administration data included non-standardised medication names, which were mapped to the World Health Organisation’s Anatomical Therapeutic Chemical Classification (ATC) [11]. In this analysis, we included systemic antibiotics (ATC code J01), but excluded methenamine hippurate (ATC code J01XX; Hiprex™), used for urinary tract infection prevention, because it is not considered to contribute to antibiotic resistance [12, 13]. Additionally, we identified antibiotics that are on the WHO Watch List. These antibiotics have high resistance potential and should be prioritised by antimicrobial stewardship programs [14]. They include: quinolones (ATC code J01M), third generation cephalosporins (ATC code J01DD), macrolides (ATC code J01FA), glycopeptides (ATC code J01XA), antipseudomonal penicillins with a beta-lactamase inhibitor (ATC codes J01CR05 and J01CR03), carbapenems (ATC code J01DH) and penems (ATC code J01DI03).

Courses of antibiotics were considered prophylaxis if they followed one of the common prophylaxis dosing regimens used in Australia [15]. These included nitrofurantoin 50-100 mg at bedtime, cefalexin 250 mg at night, and trimethoprim 150 mg at night, all for UTI prophylaxis; and trimethoprim with sulfamethoxazole 80/400–160/800 mg once daily or 160/800 mg three times a week for *Pneumocystis pneumonia* prophylaxis.

### Identification of health conditions and presence of resistant infectious organisms

We used the ACFI and free text fields to identify prevalent conditions and a history of infections that may influence antibiotic use. The ACFI provides the top three most impactful medical conditions affecting a resident’s care needs as assessed by the aged care provider, and is used for funding purposes. The free text field contained a list of each resident’s conditions. We developed algorithms [16] to flag relevant text strings that identified conditions and a history of infections that may influence antibiotic use, as well as whether the resident was a carrier of resistant infectious organisms. Importantly, the data did not allow us to reliably identify incidence and duration of infections, but only that the resident had a history of infectious conditions. The health conditions identified were dementia, chronic respiratory disease (asthma, chronic obstructive pulmonary disease), urinary incontinence; and a history of UTIs, wounds (including pressure ulcers, skin tears), and skin and soft tissue infections (SSTI). The infectious organisms that show high levels of resistance identified included methicillin-resistant *Staphylococcus aureus* (MRSA), *Clostridioides difficile*, and extended spectrum beta-lactamases (ESBL).

### Outcomes

The primary outcome of interest was antibiotic days of therapy per 1000 resident days (DOT/1000 days). Consistent with previous methods, DOT was estimated by counting each day a specific antibiotic was administered to a resident. If two different antibiotics were administered on one day, this is counted as two days (see Additional file 1). The number of days each resident was present in a RAC home was estimated using the medication administration record to count the number of days that medications were administered. For the small proportion of residents without any regular medication use (*n* = 188; 1.9%), we counted their days in the RAC home using the dates of admission and discharge.

Secondary outcomes of interest were other core indicators of antibiotic use [17–19]: percent of residents receiving at least one antibiotic course annually and number of courses per 1000 resident days (courses/1000 days). An antibiotic course was defined as the administration of a specific antibiotic over consecutive days, allowing for three-day gaps in administration (see Additional file 1). These gaps accommodate brief outages in the electronic system, disruptions in medication supply and time away from a RAC home. Lastly, we also determined the most common types of antibiotics used (based on courses) and the course duration. Outcomes of interest were generated for antibiotics overall and for antibiotics on the WHO Watch List.

### Statistical analysis

Descriptive statistics were generated for types of antibiotics used and course duration (median and interquartile range [IQR]). Plots of crude rates of DOT/1000 days per month were generated for all antibiotics, treatment and prophylaxis courses, and antibiotics on the WHO Watch List.

We applied generalised estimating equations (GEE) regression to our primary outcome DOT/1000 days to examine changes over time in antibiotic use. We used a modified Park Test to determine the appropriate distribution and link function [20]. The GEE regression (specifying a gamma distribution and a log link function, with an unstructured covariance matrix) was clustered on resident with fixed effects for RAC home to account for repeated measures of residents over time and within RAC homes. To examine temporal trends while controlling for seasonal variation in use, we used a person-month dataset (i.e. a dataset with one record for each resident who was present during each month in a RAC home), and included dummy variables for each calendar month and year as a continuous variable. The model was adjusted for resident demographics and health conditions. We also tested for interactions between age and sex, age at entry into the RAC home and age, and age at entry into the RAC home and sex. Interaction terms were removed in a stepwise manner if they did not reach a significance level of *p* ≤ 0.2 or did not behave as a confounder [21]. To assess whether there was a change in the use of antibiotics on the WHO Watch List, we included a dummy variable representing the use of a Watch List antibiotic, and an interaction term between this dummy variable and year to allow year effects to vary for these specific antibiotics. We fitted a fully adjusted “final” model and a model without resident characteristics to examine how resident characteristics changed the estimates for year. For both models, we estimated marginal effects which represent the change in DOT/1000 days associated with a change in a given independent variable from the base/reference level.

To estimate antibiotic use for each year, we modelled annual primary and secondary antibiotic use indicators using data from 2015 through 2017. The year 2014 was excluded from the annual models as data were only available for three months of that year. We used a person-year dataset and the same modelling approach as described above. However, we included a dummy variable for whether a resident was present during the months of higher antibiotic use in any given year (May to September) to control for seasonal trends.

To examine variation between RAC homes, we generated modelled annual DOT/1000 days for each RAC home for 2016, adjusted and unadjusted for resident characteristics. Caterpillar plots with these estimates were generated, with a reference line for the mean DOT/1000 days across all RAC homes. RAC homes with a DOT/1000 days estimate that changed by more than 10% after adjusting for resident factors were highlighted with the following markers: i) red, if the change was > 10% *higher* compared to the unadjusted estimate, and ii) green, if the change was > 10% *lower* compared to the unadjusted estimate.

Data management and analyses used SAS 9.4 (SAS Institute, Cary, NC) and Stata 15 (Stata Corp, College Station, TX). Since we analysed multiple outcomes that were not independent, we used a type I error rate of 0.01.

## Results

### Sample characteristics

The final dataset included 9793 unique permanent residents who were present in the RAC homes for a total of 5,230,215 resident days between 1 September 2014 and 28 September 2017. Resident characteristics are shown in Table 1.

**Table 1:** Characteristics of 9793 unique residents in 68 RAC homes, 2014–2017

Characteristics of residents^a^	2014^b^ (***n*** = 5325 residents)	2015 (n = 6528 residents)	2016 (***n*** = 6579 residents)	2017^c^ (***n*** = 5908 residents)
Total resident days	491,045	1,733,701	1,726,336	1,279,133
Females	71.1%	69.0%	67.8%	67.7%
Age, mean (SD)	85.5 (8.1)	85.8 (8.1)	85.8 (8.1)	85.9 (8.1)
Age females, mean (SD)	86.7 (7.6)	87.1 (7.7)	87.1 (7.7)	87.2 (7.8)
Age males, mean (SD)	82.5 (8.3)	83.1 (8.3)	83.1 (8.2)	83.4 (8.2)
Age at admission, mean (SD)	82.6 (8.6)	82.9 (8.5)	82.8 (8.5)	82.7 (8.6)
Comorbidities				
Dementia	56.5%	56.7%	55.1%	54.5%
Chronic respiratory disease	35.8%	35.7%	35.9%	34.4%
Urinary incontinence	39.0%	44.7%	46.3%	48.5%
History of urinary tract infection	15.0%	15.1%	15.1%	14.5%
History of wound	5.1%	5.7%	5.4%	5.3%
History of skin and soft tissue infections	5.2%	5.6%	5.7%	5.3%
History of resistant infectious organisms				
MRSA	3.8%	4.0%	3.9%	3.4%
Clostridioides difficile	0.2%	0.3%	0.3%	0.3%
ESBL	0.2%	0.2%	0.2%	0.3%
In-dwelling catheter	3.0%	3.3%	3.2%	3.0%

*SD* is standard deviation. *MRSA* is *methicillin-resistant Staphylococcus aureus*. *ESBL* is extended spectrum beta-lactamases.

^a^The characteristics are calculated for the total number of residents present in RAC homes during the study period. Thus, for categorical variables, the denominator used is the number of residents

^b^Data available for September through December in 2014.

^c^Data available for January through September in 2017.

### Most common antibiotics and course length

Over the entire study period, there were 40,549 courses of antibiotics observed, of which 809 were likely prophylaxis. The most commonly used antibiotics are shown in Table 2.

**Table 2:** Most commonly prescribed courses of antibiotics across 68 RAC homes, 2014–2017

Antibiotic	No. of courses (%)
**All courses (*n*** **= 40,549)**	
Cefalexin	13,262 (32.7)
Amoxicillin with clavulanic acid	5216 (12.9)
Trimethoprim	3903 (9.6)
Amoxicillin	3890 (9.6)
Roxithromycin	2615 (6.5)
Doxycycline	2589 (6.4)
Trimethoprim with sulfamethoxazole	1452 (3.6)
Flucloxacillin	990 (2.4)
Ciprofloxacin	954 (2.4)
Clarithromycin	910 (2.2)
**Prophylaxis courses (*n*** **= 809)**	
Cefalexin	482 (59.6)
Trimethoprim	177 (21.9)
Nitrofurantoin	150 (18.5)
**Courses of antibiotics on WHO Watch list (*n*** **= 5529)**	
Roxithromycin	2615 (47.3)
Ciprofloxacin	954 (17.3)
Clarithromycin	910 (16.5)
Norfloxacin	473 (8.6)
Erythromycin	346 (6.3)
Azithromycin	156 (2.8)
Moxifloxacin	44 (0.80)
Ceftriaxone	31 (0.56)

There were 5529 courses of antibiotics on the WHO Watch List, with roxithromycin, ciprofloxacin, and clarithromycin accounting for 81.1% of these courses (Table 2). The median duration of an antibiotic course was 7.0 days (IQR: 5.0–8.0), 7.0 days (IQR: 2.0–30.0) for prophylaxis courses, and 7.0 days (IQR: 5.0–9.0) for antibiotics on the WHO Watch List. Of all courses, 60.3% were ≤ 7 days, 32.7% were 8–14 days long, 3.4% were 15–21 days, 0.86% were 22–28 days and 2.7% were > 28 days.

### Trends in antibiotic use and key factors associated with use

**Fig. 1:**
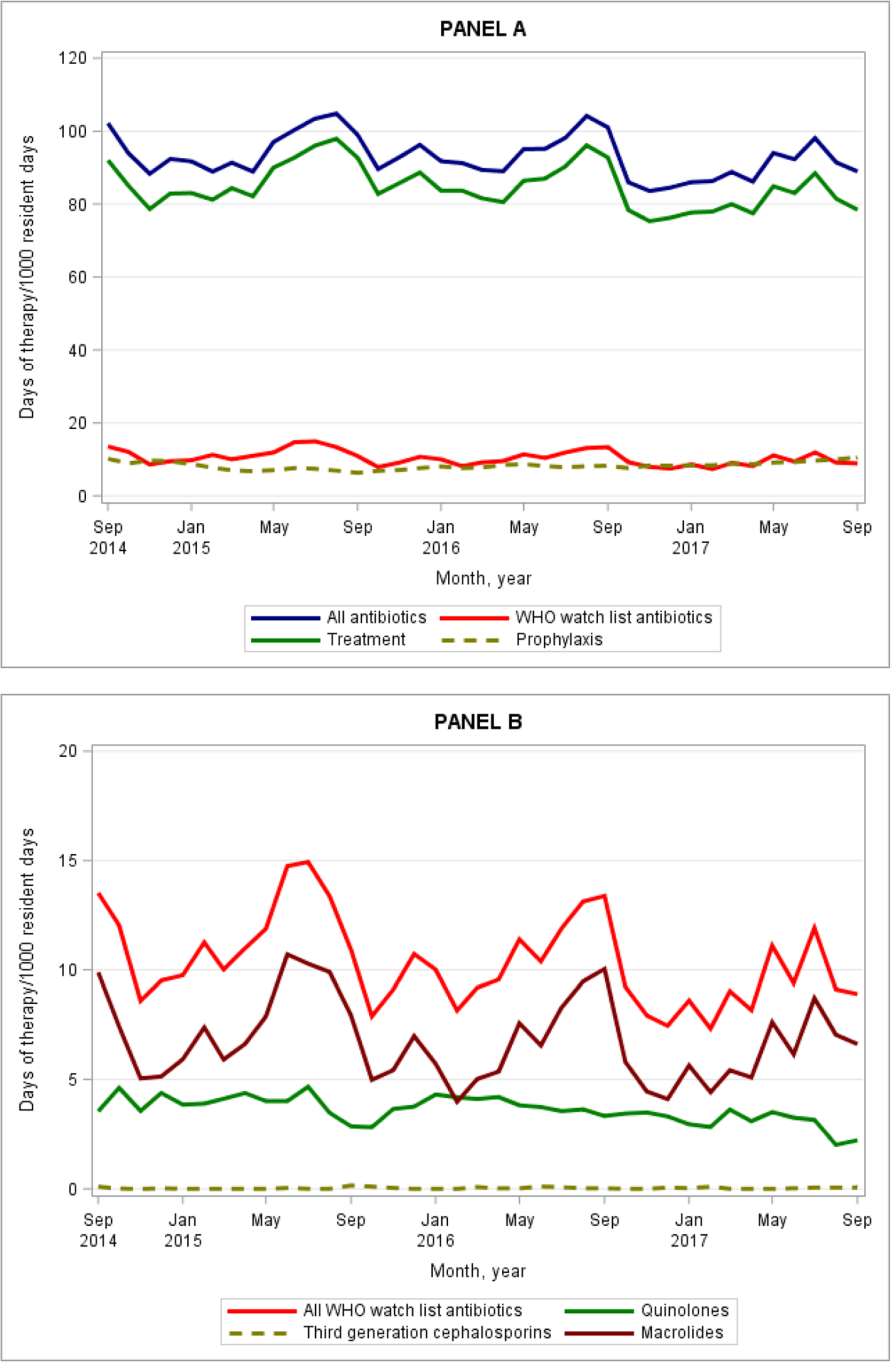
Crude monthly days of antibiotic therapy per 1000 resident days for 68 RAC homes, 2014–2017. Panel **a** includes use of all antibiotics, antibiotics on the World Health Organisation’s Watch List, and treatment and prophylaxis. Panel **b** includes antibiotics on the WHO’s Watch List overall and by antibiotic class

Figure 1 (Panel A) shows the crude monthly trends in antibiotic use (DOT/1000 resident days). There were spikes in use between May and September each year, coinciding with the winter months and influenza season. Overall antibiotic use was driven by treatment of infections, rather than prophylaxis use. Figure 1 (Panel B) shows the monthly trends in use of all WHO Watch list antibiotics and specific classes within this group. Macrolides were the dominant class used with a peak between May and September, likely for the treatment of respiratory tract infections. Use of quinolones showed less seasonal variation in use.

**Fig. 2:**
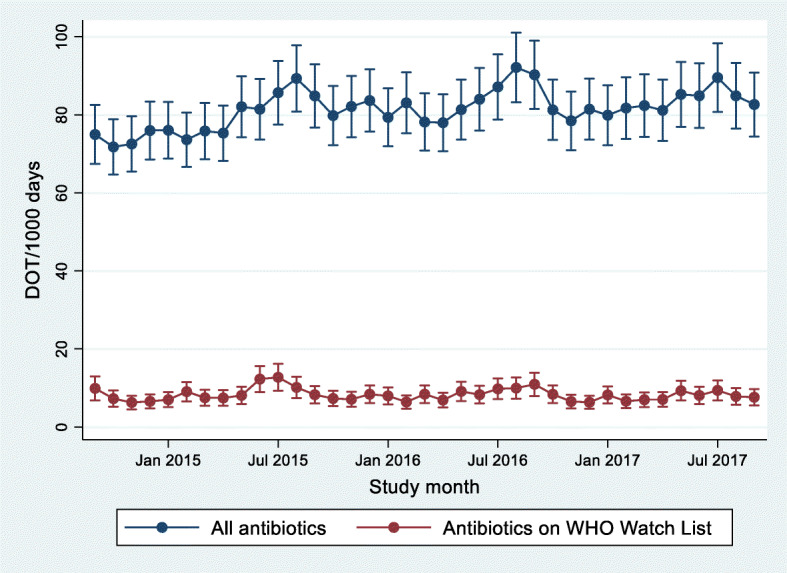
Modelled monthly estimates (and 99% confidence intervals) of days of antibiotic therapy per 1000 resident days for 68 RAC homes. Estimates are adjusted for resident characteristics, between September 2014 and September 2017

Table 3 shows the marginal effects for changes in antibiotic DOT/1000 resident days adjusted and unadjusted for resident characteristics and health conditions. Not adjusting for resident characteristics, there was an increase of 4.09 DOT/1000 days (99% CI: 1.18, 6.99) per year. Accounting for resident characteristics and comorbidities reduced this effect to be non-statistically significant at the 99% CI level (3.12 DOT/1000 days [99% CI: − 0.06, 6.29] per year). In both models, the time trend did not differ for antibiotics on the WHO Watch List (i.e. no significant interaction between WHO Watch List and year). Antibiotic use peaked in the month of August with use 12.36 DOT/1000 days (99% CI: 6.91–17.81) higher than in January. Figure 2 shows the modelled monthly antibiotic use trends for all antibiotics and antibiotics on the WHO Watch List.

Of the resident characteristics, increasing age; chronic respiratory disease; a history of UTIs, wounds and SSTIs; the presence of MRSA; and catheter use were all associated with higher antibiotic use. However, presence of dementia was associated with lower use (Table 3).

### Annual estimates of antibiotic use

Table 4 shows the modelled annual estimates of the key antibiotic use indicators, adjusted for resident characteristics and health conditions, for all antibiotics and antibiotics on the WHO Watch List (Additional file 2 shows the crude estimates). The DOT/1000 days and number of courses/1000 days did not increase significantly between 2015 and 2017. However, the annual percentage of residents with at least one antibiotic course and a WHO Watch List antibiotic course decreased significantly from 2015 to 2017.

**Table 3:** Marginal effects estimated by GEE regression of DOT/1000 resident days unadjusted and adjusted for resident characteristics and comorbidities

	Unadjusted model			Adjusted model		
	Marginal effect	99% CI	***p***-value	Marginal effect	99% CI	***p***-value
Year	4.09	1.18, 6.99	< 0.001	3.12	−0.05, 6.29	0.011
Month						
January	Reference			Reference		
February	−0.19	−3.71, 3.33	0.889	0.98	−3.01, 4.96	0.527
March	−0.67	−4.71, 3.38	0.672	−0.03	−4.53, 4.46	0.985
April	−2.08	−6.12, 1.96	0.185	−1.23	−5.52, 3.06	0.459
May	3.52	−1.06, 8.11	0.048	5.14	0.2, 10.08	0.007
June	3.95	−0.78, 8.69	0.032	5.51	0.32, 10.7	0.006
July	8.27	3.32, 13.21	0.000	9.70	4.37, 15.03	< 0.001
August	10.51	5.38, 15.63	0.000	12.36	6.91, 17.81	< 0.001
September	5.48	0.79, 10.17	0.003	7.47	2.36, 12.59	< 0.001
October	0.01	−4.65, 4.66	0.998	1.36	−3.79, 6.51	0.496
November	0.06	−4.47, 4.6	0.971	1.83	−3.25, 6.92	0.352
December	3.38	−0.78, 7.55	0.036	5.40	0.59, 10.2	0.004
WHO Watch List Antibiotic	219.56	187.14, 251.97	< 0.001	247.04	206.61, 287.47	< 0.001
WHO Watch List antibiotic x year	−0.41	−5.96, 5.14	0.849	−1.64	−7.96, 4.67	0.503
Age				2.28	0.00, 3.91	0.000
Age at admission				−0.54	−2.05, 0.97	0.358
Men				−2.48	−12.99, 8.02	0.542
Comorbidities						
Dementia				−9.93	−19.12, −0.74	0.005
Chronic respiratory disease				27.48	16.66, 38.31	< 0.001
Urinary incontinence				3.36	−4.57, 11.28	0.275
History of UTI				50.18	32.01, 68.36	< 0.001
History of wound				28.78	1.28, 56.28	0.007
History of SSTI				30.07	5.28, 54.86	0.002
Presence of resistant infectious organisms						
MRSA				54.74	15.46, 94.03	< 0.001
Clostridioides difficile				−19.86	−94.4, 54.67	0.492
ESBL				106.52	− 101.52, 314.56	0.187
In-dwelling catheter				45.83	6.72, 84.94	0.003

GEE is generalised estimating equations. DOT is days of therapy. CI is confidence interval. MRSA is methicillin-resistant *Staphylococcus aureus*. ESBL is extended spectrum beta-lactamases. Model includes fixed effects for facilities to adjust for clustering within facilities. Marginal effects can be interpreted as the change in DOT/1000 resident days associated with a change in a given independent variable from the base/reference level, independent of all the other covariates in the model

### Variation in antibiotic use between RAC homes

**Fig. 3:**
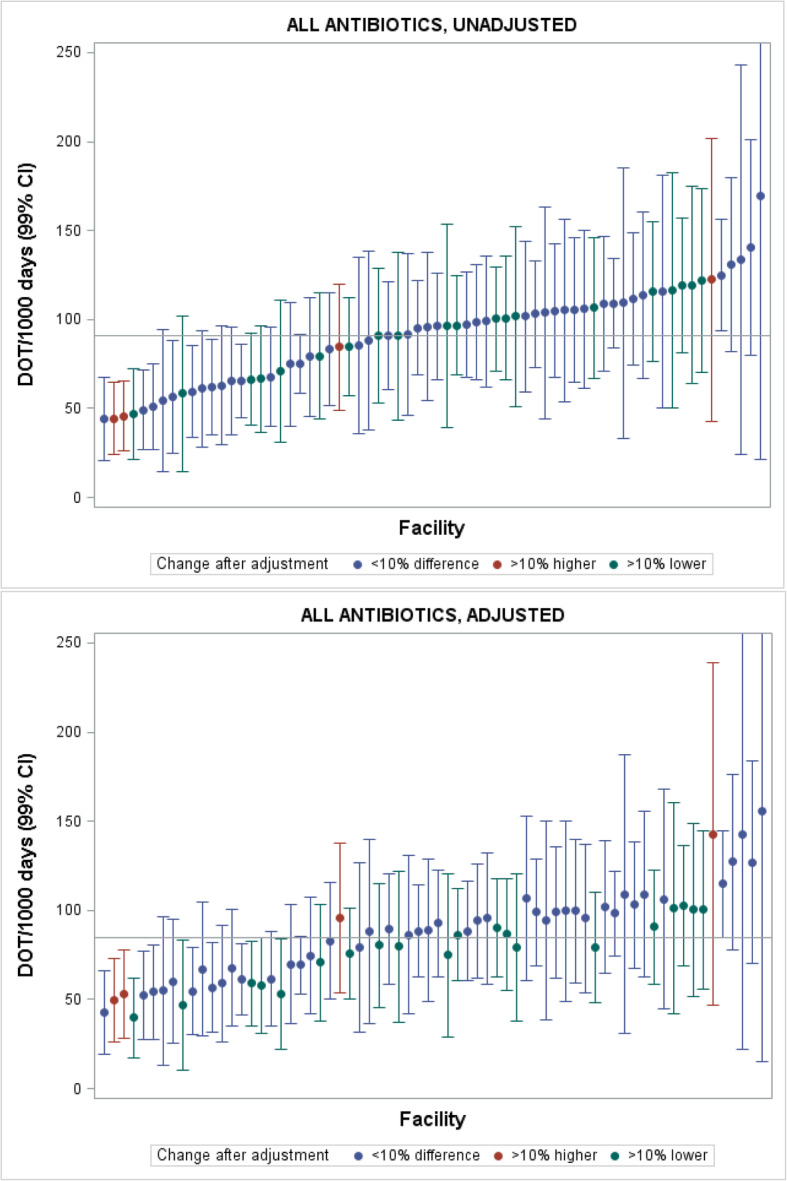
Caterpillar plots of modelled estimates of days of antibiotic therapy per 1000 resident days for 68 RAC homes unadjusted and adjusted for resident characteristics and health conditions, 2016. RAC homes with red markers are ones where the DOT/1000 days is more than 10% higher than the home’s unadjusted estimate after adjusting for resident characteristics and health conditions. Homes with green markers are ones where the DOT/1000 days is more than 10% lower after adjusting for resident characteristics and health conditions. Homes with blue markers are ones where there is a less than 10% change in DOT/1000 days after adjustment for resident characteristics and health conditions

The caterpillar plots show wide variation between RAC homes in antibiotic DOT/1000 days in 2016 (Fig. 3). Unadjusted for resident characteristics and conditions, use ranged between 44.0 and 169.2 DOT/1000 days; and between 42.6 and 155.5 DOT/1000 days after adjustment. Four RAC home estimates of antibiotic use increased by over 10% and 20 decreased by over 10% after adjustment for resident factors.

## Discussion

Using electronic health record data, we examined antibiotic use trends in 68 RAC homes in Australia between 2014 and 2017. Our analysis suggests that antibiotic use may be increasing in RAC, as measured by DOT/1000 days, in these RAC homes between September 2014 and September 2017. Over the same period, there was no change in the DOT/1000 days of antibiotics on the WHO Watch List, i.e., those with high resistance potential. However, there was a small statistically significant decrease in the percentage of residents with at least one antibiotic course annually (68.4 to 63.4%; Table 4). We also identified wide variation in antibiotic use between RAC homes that was not explained by resident factors, which is suggestive of differences in antibiotic prescribing practices in RAC homes.

National antibiotic consumption in Australia has been decreasing steadily since 2015 [5]. In contrast, our analysis using conservative 99% confidence intervals suggests an increase in use, or at best no change in overall antibiotic use in RAC between 2014 and 2017. An increase of 3.12 DOT/1000 days (99% CI: − 0.05, 6.29) is roughly equivalent to 1 extra day of antibiotic therapy per resident annually. High levels of inappropriate antibiotic use in RAC in Australia have been reported. In 2019, the point prevalence AURA surveys estimated that only 14.7, 18.4 and 3.6% of antibiotics for respiratory tract infections, SSTI, and urinary tract infections, respectively, were appropriate when assessed against the McGeer criteria [6]. While there have been coordinated effort to reduce antibiotic use in the community in Australia over some decades, our study suggests these have not had an impact on antibiotic use in RAC. Thus, there is a clear need for targeted strategies to improve antibiotic use in the residential aged care population.

The trend in use of antibiotics on the WHO Watch List did not differ from that of the trend for overall antibiotic use during study period. Macrolides were the most commonly used antibiotic in this group, with increases during influenza season. Further examination of the reason for use of macrolides is warranted to understand how their use can be limited. Importantly, our analysis showed that residents who used a WHO Watch List antibiotic (compared to those who did not) had substantially higher use of antibiotics overall (247 DOT/1000 days higher), which equates approximately to 90 days among every 3 residents annually. Ciprofloxacin was the second most commonly used course of WHO Watch List antibiotics. In Australia, ciprofloxacin use is reserved for gram-negative infections resistant to other antibiotics and/or for use in immunocompromised individuals, which may account for why residents on antibiotics on the WHO Watch List used antibiotics at higher rates.

We selected DOT/1000 days as our primary indicator for antibiotic use as it accounts for both the frequency of antibiotic courses and the length of each course, making it the most sensitive measure of antibiotic use. However, we also estimated other commonly used indicators of antibiotic use to enable comparisons with other studies. While we found no change in the DOT/1000 days during the study period, there was a decrease in the percentage of residents with at least one antibiotic course in 2017 compared to 2015 (Table 4). This discrepancy suggests that residents are initiated on antibiotics less often, but are likely staying on them for longer. An increase in course length is a concern since cumulative exposure to antibiotics has an important role in the development of resistance and complications [22, 23]. Guidelines on course length in recent years have changed due to mounting evidence that shorter treatment is just as effective as longer treatment for the great majority of infections [24, 25]. However, in Australia, antibiotics are supplied in standard pack sizes and with an allowance for a repeat supply, which results in the supply of doses in excess of the recommended durations for most infections [26]. Furthermore, documentation of course duration or review date on antimicrobial courses in RAC is poor, with only 40% containing this information [6]. Thus, antibiotic course length should be a target for antimicrobial stewardship activities in RAC in Australia.

We found wide variation in antibiotic use rates between RAC homes. We attempted a priori to account for key resident factors that could explain antibiotic use and that were measurable with the data available. However, these did not fully explain the variation between RAC homes (Fig. 3), indicating that unmeasured factors are also driving this variability. The literature suggests that, aside from other clinical conditions affecting residents, doctor and RAC home level factors, as well as resident and family expectations, around antibiotic use may be responsible for variation between RAC homes [27–29]. Our results suggest that antimicrobial stewardship activities could be more intensive and targeted at RAC homes that have higher rates of antibiotic use. Furthermore, understanding why some RAC homes had low rates of antibiotic use could also inform intervention development.

Our study demonstrates important methodological considerations for future studies. Our modelled estimates of DOT/1000 days were different to the crude estimates (see Table 4 and Additional file 2). Additional file 3 shows the year effect estimated from consecutive models with added adjustments for clustering and independent variables. Model 5 is the final model adjusted for all independent variables (full results in Table 3). Model 1, which does not account for repeated measures in residents and clustering in RAC homes, shows a significant decrease of 3.05 DOT/1000 days (99% CI: − 4.77,-1.34; *p* < 0.001) each year. Accounting for repeated measures within residents in Model 2 changes the direction of this effect (4.08, 99% CI: 1.17, 6.99; *p* < 0.001), and subsequent adjustments reduce this effect. We conducted a post-hoc analysis examining whether there were differences in our cohort over time, which revealed that residents were entering RAC homes at older ages over time (Additional file 4). This effect was more pronounced for men compared to women (see Additional file 4). Our modelling shows that antibiotic use increases with age (Table 3) and we have further illustrated this with Additional file 5 which shows that antibiotic use is higher for older residents. In summary, our data show that residents entering RAC homes at older ages are treated more aggressively with antibiotics compared to their younger counterparts. This effect is more pronounced for residents entering RAC homes in the later years of our study period (2017 as compared to 2015). Further investigation into why this is the case is warranted.

**Table 4:** Modelled^a^ estimates of annual antibiotic use (and 99% CIs) across 68 RAC homes, 2015–2017

	2015 (***n*** = 6528)	2016 (***n*** = 6579)	2017 (n = 5908)
**All antibiotics**			
Days of therapy/1000 resident days	81.57 (75.38, 87.75)	83.30 (77.95, 88.64)	85.06 (78.56, 91.57)
Number of courses/1000 resident days	7.60 (7.19, 8.01)	7.81 (7.49, 8.12)	8.01 (7.57, 8.46)
Percent of residents with at least one course of antibiotics	68.39 (66.93, 69.85)	65.94 (64.90, 67.00)	63.44 (61.87, 65.01)
**WHO Watch List antibiotics**			
Days of therapy/1000 resident days	8.78 (7.61, 9.92)	8.23 (7.46, 9.00)	7.73 (6.69, 8.77)
Number of courses/1000 resident days	1.07 (0.94, 1.20)	0.99 (0.90, 1.08)	0.92 (0.80, 1.03)
Percent of residents with at least one course of antibiotics	17.02 (15.85, 18.19)	15.17 (14.39, 15.95)	13.46 (12.38, 14.55)

*CI* is confidence interval.

^a^Modelled using generalised estimating equations with fixed effects for RAC home, resident presence in winter months (May to September). Models are adjusted for resident demographics and comorbidities

Our estimates of antibiotic use are broadly comparable to those reported in the few Australian studies on this topic (see Additional file 6). One study in two Australian RAC homes reported that 79% of residents had an antibiotic during a 278 day period [30]. Cefalexin was the most commonly prescribed antibiotic in two studies and the AURA point prevalence surveys of 2015–2018 [6, 30–32]. Only one other study measured antibiotic use in DOT/1000 resident days and reported use in two RAC homes over two periods in 2012 (Sep-Nov) and 2013 (May-Jul) before and after an intervention: 62.0 and 49.6 DOT/1000 days [31]. This was notably lower than our estimate of 81.57 (99% CI: 75.38–87.75) DOT/1000 days in our sample in 2015. However, in our sample some RAC homes had rates of use below 50 DOT/1000 days. Comparison of our estimates to those reported in international studies further illustrates the variation in use between RAC homes [33]. Understanding the reasons behind this variation is central to informing antimicrobial stewardship in this setting.

To our knowledge this is the first and largest Australian study of longitudinal trends of antibiotic use in RAC using contemporary electronic health record data. The study has several strengths, including using a regression approach that accounted for repeated measures within RAC homes, and examining important resident characteristics. We have also generated detailed information on antibiotic use in RAC, including testing for trends over time which fills an important knowledge gap. However, there are some limitations to our study. We used data from one provider with facilities across two states in Australia. It is possible that antibiotic use differs in RAC from other providers or in government RAC or in other states, which may limit the generalisability of our results. Our previous work with these data has shown that our sample is demographically similar to the national RAC population [10, 16]. Another limitation of our data was that we did not have incident diagnosis information, or data on resident signs and symptoms, which prohibited assessments of appropriateness of therapy. Lastly, further years of data would be required to confirm the long-term trend in antibiotics use in RAC.

## Conclusions

Our analysis has highlighted important target areas for antimicrobial stewardship programs in RAC in Australia. Although implementation of electronic records with medication management is not universal in Australian RAC, it is likely that uptake will increase in coming years. We have provided a model for the use of electronic record data for timely surveillance of antibiotic use in RAC using multiple indicators that could be expanded to cover a large number of facilities.

## Supplementary information

**Additional file 1.**

**Additional file 2.**

**Additional file 3.**

**Additional file 4.**

**Additional file 5.**

**Additional file 6.**

## Data Availability

The datasets used in this study are not publicly available and were obtained under an agreement with the aged care provider.

## Abbreviations

ACFI: Aged Care Funding Instrument
ATC: Anatomical Therapeutic Chemical Classification
AURA: Antimicrobial Use and Resistance in Australia
CI: Confidence interval
DOT: Days of therapy
ESBL: Extended spectrum beta-lactamases
GEE: Generalised estimating equations
MRSA: Methicillin-resistant *Staphylococcus aureus*
OECD: Organisation for Economic Co-operation and Development
RAC: Residential aged care
SSTI: Skin and soft tissue infection
UTI: Urinary tract infection
WHO: World Health Organisation

## References

[1] World Health Organisation. Antibiotic resistance - a threat to global health security: World Health Organization; 2013. Available from: http://www.who.int/antimicrobial-resistance/events/wha66_side_event/en/.

[2] Marra F, McCabe M, Sharma P (2017). Utilization of antibiotics in long-term care facilities in British Columbia, Canada. J Am Med Dir Assoc.

[3] Thornley T, Ashiru-Oredope D, Normington A (2019). Antibiotic prescribing for residents in long-term-care facilities across the UK. J Antimicrob Chemother.

[4] van Buul LW, van der Steen JT, Veenhuizen RB (2012). Antibiotic use and resistance in long term care facilities. J Am Med Dir Assoc.

[5] Australian Commission on Safety and Quality in Health Care, AURA 2019 (2019). Third Australian report on antimicrobial use and resistance in human health. Australian Commission on Safety and Quality in Health Care.

[6] National Centre for Antimicrobial Stewardship, Australian Commission on Safety and Quality in Health Care (2019). Antimicrobial prescribing and infections in Australian aged care homes: results of the 2018 aged care National Antimicrobial Prescribing Survey. Australian Commission on Safety and Quality in Health Care.

[7] Mavromaras K, Knight G, Isherwood L, et al. 2016 National Aged Care Workforce Census and survey – the aged care workforce: Department of Health - Government of Australia; 2017. Available from: https://www.gen-agedcaredata.gov.au/www_aihwgen/media/Workforce/The-Aged-Care-Workforce-2016.pdf.

[8] Lind KE, Jorgensen ML, Gary LC (2019). Anti-osteoporosis medication use in a high fracture-risk population: contemporary trends in Australian residential aged care facilities.

[9] Lind KE, Gray LC, Raban MZ, Georgiou A, Westbrook JI (2019). Antidementia medication use by aged care facility residents with dementia. Int J Geriatr Psychiatry.

[10] Pont LG, Raban MZ, Jorgensen ML (2018). Leveraging new information technology to monitor medicine use in 71 residential aged care facilities: variation in polypharmacy and antipsychotic use. International J Qual Health Care.

[11] WHO Collaborating Centre for Drug Statistics Methodology. *ATC/DDD Index 2020*. [cited 24 April 2020]; Available from: https://www.whocc.no/atc_ddd_index/.

[12] Lee BS, Bhuta T, Simpson JM (2012). Methenamine hippurate for preventing urinary tract infections. Cochrane Database Syst Rev.

[13] Yang B, Blick C, Foley S (2020). Avoiding antibiotics in the management of recurrent UTIs in women: what are our options?. J Clin Urol.

[14] World Health Organisation (2019). WHO releases the 2019 AWaRe classification antibiotics.

[15] Australian Medicines Handbook. 2019, Adelaide: Australian Medicines Handbook Pty Ltd.

[16] Lind KE, Raban MZ, Brett L, et al. Measuring the prevalence of 60 health conditions in older Australians in residential aged care with electronic health records: a retrospective dynamic cohort study. 2020. Pre-print at: https://www.researchsquare.com/article/rs-11978/v1. 10.1186/s12963-020-00234-z PMC7545887 33032628

[17] Centers for Disease Control and Prevention. The core elements of antibiotic stewardship for nursing homes. US Department of Health and Human Services, CDC; 2015. Available from: https://www.cdc.gov/longtermcare/pdfs/core-elements-antibiotic-stewardship.pdf.

[18] European Centre for Disease Prevention and Control. Antimicrobial consumption - annual epidemiological report for 2017: European Centre for Disease Control; 2018. Available from: https://www.ecdc.europa.eu/sites/portal/files/documents/AER_for_2017-antimicrobial-consumption.pdf.

[19] Public Health Agency of Canada. Canadian antimicrobial resistance surveillance system - update 2018: Public Health Agency of Canada; 2019. Available from: https://www.canada.ca/content/dam/phac-aspc/documents/services/publications/drugs-health-products/canadian-antimicrobial-resistance-surveillance-system-2018-report-executive-summary/pub1-eng.pdf.

[20] Park RE (1966). Estimation with Heteroscedastic error terms. Econometrica..

[21] Mickey RM, Greenland S (1989). The impact of confounder selection criteria on effect estimation. Am J Epidemiol.

[22] Chatterjee A, Modarai M, Naylor NR (2018). Quantifying drivers of antibiotic resistance in humans: a systematic review. Lancet Infect Dis.

[23] Stevens V, Dumyati G, Fine LS (2011). Cumulative antibiotic exposures over time and the risk of Clostridium difficile infection. Clin Infect Dis.

[24] Llewelyn MJ, Fitzpatrick JM, Darwin E, et al. The antibiotic course has had its day. BMJ. 2017;358:j3418. 10.1136/bmj.j3418 28747365

[25] Spellberg B (2016). The new antibiotic mantra-"shorter is better". JAMA Intern Med.

[26] McGuire TM, Smith J, Del Mar C (2015). The match between common antibiotics packaging and guidelines for their use in Australia. Aust N Z J Public Health.

[27] Bennett N, Imam N, James R (2018). Prevalence of infections and antimicrobial prescribing in Australian aged care facilities: evaluation of modifiable and nonmodifiable determinants. Am J Infect Control.

[28] Daneman N, Campitelli MA, Giannakeas V (2017). Influences on the start, selection and duration of treatment with antibiotics in long-term care facilities. CMAJ Can Med Assoc J.

[29] Lim CJ, Kwong MW, Stuart RL (2014). Antibiotic prescribing practice in residential aged care facilities--health care providers' perspectives. Med J Aust.

[30] Cowan RU, Kishan D, Walton AL (2016). Cleaning, resistant bacteria, and antibiotic prescribing in residential aged care facilities. Am J Infect Control.

[31] Stuart RL, Orr E, Kotsanas D (2015). A nurse-led antimicrobial stewardship intervention in two residential aged care facilities. Healthcare Infection.

[32] Smith M, Atkins S, Worth L (2013). Infections and antimicrobial use in Australian residential aged care facilities: a comparison between local and international prevalence and practices. Australian Health Rev.

[33] Daneman N, Bronskill SE, Gruneir A (2015). Variability in antibiotic use across nursing homes and the risk of antibiotic-related adverse outcomes for individual residents. JAMA Intern Med.

