# Supplementary material for: Trends, determinants and differences in antibiotic use in 68 residential aged care homes in Australia, 2014–2017: a longitudinal analysis of electronic health record data
Source: BMC Health Serv Res. 2020 Sep 18;20:883. doi: 10.1186/s12913-020-05723-3 (PMC7501612; doi:10.1186/s12913-020-05723-3)
Supplement: Supplementary file 1 — Additional file 1. [file 12913_2020_5723_MOESM1_ESM.docx]

**Additional file 1**

**Figure: Example of measuring days of therapy (DOT) for two different residents**

In example 1, one antibiotic is administered for 7 days, resulting in a total of 7 DOT. In example 2, two different antibiotics are observed for 7 and 5 days each, resulting in a total of 12 DOT.

**Figure: Example of identification of courses for three different residents**

In example 1, one antibiotic is administered over one 5 days course. In example 2, there are two courses of different antibiotics. In example 3, there is one course of a single antibiotic, with a three-day break in administration.
