# Supplementary material for: Trends, determinants and differences in antibiotic use in 68 residential aged care homes in Australia, 2014–2017: a longitudinal analysis of electronic health record data
Source: BMC Health Serv Res. 2020 Sep 18;20:883. doi: 10.1186/s12913-020-05723-3 (PMC7501612; doi:10.1186/s12913-020-05723-3)
Supplement: Supplementary file 2 — Additional file 2. [file 12913_2020_5723_MOESM2_ESM.docx]

**Additional file 2**

Table: Crude estimates of annual antibiotic use across 68 RACFs, 2015-2017

|  | **2015**  **(n=6528)** | **2016**  **(n=6579)** | **2017***  **(n=5908)** |
| --- | --- | --- | --- |
| **All antibiotics** |  |  |  |
| Days of therapy/1000 resident days, mean | 100.4 | 98.0 | 93.8 |
| Number of courses/1000 resident days, mean | 9.7 | 9.2 | 8.9 |
| Residents who were on an antibiotic, percent | 66.6 | 65.4 | 62.2 |
| **WHO Watch List antibiotics** |  |  |  |
| Days of therapy/1000 resident days, mean | 11.9 | 10.9 | 9.9 |
| Number of courses/1000 resident days, mean | 1.37 | 1.23 | 1.17 |
| Residents who were on an antibiotic, percent | 19.0 | 17.9 | 15.2 |

*Data available for January through September in 2017.
