# Supplementary material for: Trends, determinants and differences in antibiotic use in 68 residential aged care homes in Australia, 2014–2017: a longitudinal analysis of electronic health record data
Source: BMC Health Serv Res. 2020 Sep 18;20:883. doi: 10.1186/s12913-020-05723-3 (PMC7501612; doi:10.1186/s12913-020-05723-3)
Supplement: Supplementary file 3 — Additional file 3. [file 12913_2020_5723_MOESM3_ESM.docx]

**Additional file 3**

Table: Marginal effect for year estimated by stepped GEE regression models with days of therapy/1000 resident days as outcome

|  | **Model 1 adjusted for seasonal variation** | **Model 2 adds adjustment for repeated measures within residents** | **Model 3 adds adjustment for clustering within facilities** | **Model 4 adds adjustment for resident factors** | **Model 5 adds adjustment for age at admission including interaction term** |
| --- | --- | --- | --- | --- | --- |
| Year effect | -3.05 | 4.08 | 3.75 | 3.35 | 3.12 |
| 99% CI | -4.77, -1.34 | 1.17, 6.99 | 0.80, 6.71 | 0.29, 6.41 | -0.06, 6.30 |
| p-value | 0.000 | 0.000 | 0.001 | 0.005 | 0.011 |

GEE is generalised estimating equations. DOT is days of therapy per 1000 resident days. Model 1 includes month variables to smooth seasonal variation and year. Model 2 adds an adjustment for repeated measures within residents. Model 3 adds fixed effects for facilities to adjust for clustering within facilities. Model 4 adds resident demographic and health condition variables. Model 5 is the final model and adjusts age at admission, including interaction term for age at admission and age.
