# Supplementary material for: Trends, determinants and differences in antibiotic use in 68 residential aged care homes in Australia, 2014–2017: a longitudinal analysis of electronic health record data
Source: BMC Health Serv Res. 2020 Sep 18;20:883. doi: 10.1186/s12913-020-05723-3 (PMC7501612; doi:10.1186/s12913-020-05723-3)
Supplement: Supplementary file 4 — Additional file 4. [file 12913_2020_5723_MOESM4_ESM.docx]

**Additional file 4**

**Figure: Modelled* age at entry into facility by the year of entry (post-hoc analysis)**

*Modelled using generalised estimating equations with age at admission as the dependent variable to examine change in the characteristics of the residents in our population.

Note: Data in this figure include the entire cohort from which the sample for the primary analysis was a subset of, i.e. includes residents entering facilities for short-term respite care. Age at entry into facility was modelled as a function of year entered, gender, and whether the resident was entering for respite care, and included interactions between these variables.

This figure shows that residents entering facilities in more recent years are older, with this effect differing based on gender.
