# Supplementary material for: Trends, determinants and differences in antibiotic use in 68 residential aged care homes in Australia, 2014–2017: a longitudinal analysis of electronic health record data
Source: BMC Health Serv Res. 2020 Sep 18;20:883. doi: 10.1186/s12913-020-05723-3 (PMC7501612; doi:10.1186/s12913-020-05723-3)
Supplement: Supplementary file 5 — Additional file 5. [file 12913_2020_5723_MOESM5_ESM.docx]

**Additional file 5**

**Figure S3: Modelled annual days of antibiotic therapy per 1000 resident days (DOT/1000 days) for residents entering residential aged care at 80 and 90 years old.**

This graph demonstrates the different antibiotic use rates based on current age for residents entering facilities at older ages as compared to those entering at younger ages. It was plotted from the primary model (marginal effects shown in Table 3).
