# Supplementary material for: Trends, determinants and differences in antibiotic use in 68 residential aged care homes in Australia, 2014–2017: a longitudinal analysis of electronic health record data
Source: BMC Health Serv Res. 2020 Sep 18;20:883. doi: 10.1186/s12913-020-05723-3 (PMC7501612; doi:10.1186/s12913-020-05723-3)
Supplement: Supplementary file 6 — Additional file 6. [file 12913_2020_5723_MOESM6_ESM.docx]

**Additional file 6**

Table S3: Summary antibiotic use rates (period prevalence and DOT/1000 days) reported by studies measuring antibiotic use in residential aged care facilities

| **Study** | **Country** | **Number of facilities** | **Time period** | **% of residents on an antibiotic** |
| --- | --- | --- | --- | --- |
| **Outcome: Percent of residents on at least one antibiotic** | | | | |
| Present study | Australia | 68 | Jan-Dec 2017 | 63.44 (99% CI: 61.87, 65.01) |
| Cowan, 2016 (1) | Australia (Victoria) | 2 | 278 days in 2014 | 79% |
| Daneman, 2013 (2) | Canada | 630 | Jan-Dec 2010 | 74.8% |
| Daneman, 2017 (3) | Canada | >600 | Jan-Dec 2014 | 55% |
| Gillespie, 2015 (4) | UK | 10 | Nov 2010 to Mar 2012 | 73.7% |
| **Outcome: DOT/1000 days** | | | | |
| Present study | Australia | 68 | 2017 | 85.06 (99% CI: 78.56, 91.57) |
| Present study | Australia | 68 | 2015 | 81.57 (99% CI: 75.38, 87.75) |
| Daneman, 2015 (5) | Canada | 607 | Jan 2010 – Dec 2011 | 54.6 |
| Jump, 2012 (6) | US | 1 | Jul 2006 – Jun 2009 | 175.1 |
| Jump, 2012 (6) | US | 1 | Jul 2009 – Dec 2010 | 122.3 |
| Mylotte, 2005 (7) | US | 11 | 2003 | 115.6 |
| Stuart, 2015 (8) | Australia (Victoria) | 2 | Sep-Nov 2012 | 62.0 |
| Stuart, 2015 (8) | Australia (Victoria) | 2 | May-Jul 2013 | 49.6 |

DOT/1000 days is days of therapy per 1000 resident days

**References**

1. Cowan RU, Kishan D, Walton AL, Sneath E, Cheah T, Butwilowsky J, et al. Cleaning, resistant bacteria, and antibiotic prescribing in residential aged care facilities. American Journal of Infection Control. 2016;44(3):e19-21.

2. Daneman N, Gruneir A, Bronskill SE, Newman A, Fischer HD, Rochon PA, et al. Prolonged antibiotic treatment in long-term care: role of the prescriber. JAMA Intern Med. 2013;173(8):673-82.

3. Daneman N, Campitelli MA, Giannakeas V, Morris AM, Bell CM, Maxwell CJ, et al. Influences on the start, selection and duration of treatment with antibiotics in long-term care facilities. CMAJ. 2017;189(25):E851-E60.

4. Gillespie D, Hood K, Bayer A, Carter B, Duncan D, Espinasse A, et al. Antibiotic prescribing and associated diarrhoea: a prospective cohort study of care home residents. Age Ageing. 2015;44(5):853-60.

5. Daneman N, Bronskill SE, Gruneir A, Newman AM, Fischer HD, Rochon PA, et al. Variability in Antibiotic Use Across Nursing Homes and the Risk of Antibiotic-Related Adverse Outcomes for Individual Residents. JAMA Intern Med. 2015;175(8):1331-9.

6. Jump RL, Olds DM, Seifi N, Kypriotakis G, Jury LA, Peron EP, et al. Effective antimicrobial stewardship in a long-term care facility through an infectious disease consultation service: keeping a LID on antibiotic use. Infection control and hospital epidemiology. 2012;33(12):1185-92.

7. Mylotte JM, Keagle J. Benchmarks for antibiotic use and cost in long-term care. Journal of the American Geriatrics Society. 2005;53(7):1117-22.

8. Stuart RL, Orr E, Kotsanas D, Gillespie EE. A nurse-led antimicrobial stewardship intervention in two residential aged care facilities. Healthcare Infection. 2015;20(1):4-6.
